# Correction to “Maternal vitamin D status in relation to infant BMI growth trajectories up to 2 years of age in two prospective pregnancy cohorts”

**DOI:** 10.1002/osp4.664

**Published:** 2023-02-09

In Amberntsson et al.,^1^ there was some incorrect information regarding the analyses of 25‐hydroxyvitamin D in the Section 2.3 *Blood sample analysis*.

The below information was incorrect:

For all women, 25OHD concentrations were measured by liquid chromatography tandem‐mass spectrometry (LC‐MS/MS). The LC‐MS/MS measures both 25OHD2 and 25OHD3 and the sum of these two is regarded as the exposure 25OHD. In MoBa, 25OHD was analyzed in plasma at the National Institute for Health and Welfare, Helsinki, Finland. In GraviD, 25OHD was analyzed in serum at the clinical chemistry laboratory in Skåne, Sweden. Both laboratories were certified by the Vitamin D External Quality Assessment Scheme.

The correct information is as follows:

In MoBa, plasma 25OHD concentrations were analyzed using the high through‐put automated chemiluminescent microparticle immunoassay Architect ci8200 system (Abbott Laboratories, Abbott Park, IL, USA). Samples were analyzed at the National Institute for Health and Welfare, Helsinki, Finland. In GraviD, serum 25OHD concentrations were measured by liquid chromatography–tandem‐mass spectrometry (LC‐MS/MS). Samples were analyzed in serum at the clinical chemistry laboratory in Skåne, Sweden. Both 25OHD assay methods measures 25OHD2 and 25OHD3 and the sum of these two is regarded as the exposure 25OHD. Both laboratories were certified by the Vitamin D External Quality Assessment Scheme.

We apologize for this error.
